# Histochemical Evidence for Nitrogen-Transfer Endosymbiosis in Non-Photosynthetic Cells of Leaves and Inflorescence Bracts of Angiosperms

**DOI:** 10.3390/biology11060876

**Published:** 2022-06-07

**Authors:** April Micci, Qiuwei Zhang, Xiaoqian Chang, Kathryn Kingsley, Linsey Park, Peerapol Chiaranunt, Raquele Strickland, Fernando Velazquez, Sean Lindert, Matthew Elmore, Philip L. Vines, Sharron Crane, Ivelisse Irizarry, Kurt P. Kowalski, David Johnston-Monje, James F. White

**Affiliations:** 1 Department of Plant Biology, Rutgers University, New Brunswick, NJ 08901, USA; qz93@scarletmail.rutgers.edu (Q.Z.); xc219@scarletmail.rutgers.edu (X.C.); klk158@sebs.rutgers.edu (K.K.); lp700@scarletmail.rutgers.edu (L.P.); pc702@rutgers.edu (P.C.); rfs109@scarletmail.rutgers.edu (R.S.); flv7@scarletmail.rutgers.edu (F.V.); slindert@scarletmail.rutgers.edu (S.L.); matthew.elmore@rutgers.edu (M.E.); plv19@sebs.rutgers.edu (P.L.V.); 2 Department of Biochemistry and Microbiology, Rutgers University, New Brunswick, NJ 08901, USA; sharron.crane@rutgers.edu; 3 School of Health and Sciences, Universidad del Sagrado Corazón, San Juan 00914, Puerto Rico; ivelisse.irizarry@sagrado.edu; 4 US Geological Survey Great Lakes Science Center, Ann Arbor, MI 48105, USA; kkowalski@usgs.gov; 5 Max Planck Tandem Group in Plant Microbial Ecology, Universidad del Valle, Cali 760043, Colombia; david.johnston@correounivalle.edu.co

**Keywords:** endophytes, histochemistry, nitrate, nitrogen-use efficiency, trichomes, nuclei, phyllosphere

## Abstract

**Simple Summary:**

We used light and confocal microscopy to visualize bacteria in leaf and bract cells of more than 30 species in 18 families of seed plants. We detected chemical exchanges between intracellular bacteria and plant cells. We found that endophytic bacteria that show evidence of the transfer of nitrogen to plants are present in non-photosynthetic cells of leaves and bracts of diverse plant species. Nitrogen transfer from bacteria was observed in epidermal cells, various filamentous and glandular trichomes, and other non-photosynthetic cells. The most efficient of the nitrogen-transfer endosymbioses were seen to involve glandular trichomes, as seen in hops (*Humulus lupulus*) and hemp (*Cannabis sativa*). Trichome chemistry is hypothesized to function to scavenge oxygen around bacteria to facilitate nitrogen fixation.

**Abstract:**

We used light and confocal microscopy to visualize bacteria in leaf and bract cells of more than 30 species in 18 families of seed plants. Through histochemical analysis, we detected hormones (including ethylene and nitric oxide), superoxide, and nitrogenous chemicals (including nitric oxide and nitrate) around bacteria within plant cells. Bacteria were observed in epidermal cells, various filamentous and glandular trichomes, and other non-photosynthetic cells. Most notably, bacteria showing nitrate formation based on histochemical staining were present in glandular trichomes of some dicots (e.g., *Humulus lupulus* and *Cannabis sativa*). Glandular trichome chemistry is hypothesized to function to scavenge oxygen around bacteria and reduce oxidative damage to intracellular bacterial cells. Experiments to assess the differential absorption of isotopic nitrogen into plants suggest the assimilation of nitrogen into actively growing tissues of plants, where bacteria are most active and carbohydrates are more available. The leaf and bract cell endosymbiosis types outlined in this paper have not been previously reported and may be important in facilitating plant growth, development, oxidative stress resistance, and nutrient absorption into plants. It is unknown whether leaf and bract cell endosymbioses are significant in increasing the nitrogen content of plants. From the experiments that we conducted, it is impossible to know whether plant trichomes evolved specifically as organs for nitrogen fixation or if, instead, trichomes are structures in which bacteria easily colonize and where some casual nitrogen transfer may occur between bacteria and plant cells. It is likely that the endosymbioses seen in leaves and bracts are less efficient than those of root nodules of legumes in similar plants. However, the presence of endosymbioses that yield nitrate in plants could confer a reduced need for soil nitrogen and constitute increased nitrogen-use efficiency, even if the actual amount of nitrogen transferred to plant cells is small. More research is needed to evaluate the importance of nitrogen transfer within leaf and bract cells of plants.

## 1. Introduction

Early in the evolution of land plants, microbial symbiosis was essential to terrestrial colonization [1,2]. Plants are thought to have primarily benefitted from these symbioses by receiving help from microbes in acquiring nutrients and resisting the stresses of life on land [1,3]. Through symbiosis, plants acquired nutrients and adapted to stress. Nitrogen is a critically important nutrient that is used by plants to build proteins and other organic molecules for primary and secondary metabolism [4]. Nitrogen concentration is limited in many soils and may be insufficient to support plant growth [5,6,7,8]. It is believed that the first symbioses helped plants absorb nutrients when mycorrhizal fungi began transmitting soil-derived nutrients to roots more than 435 million years ago [1], and this relationship continues today in about 90% of plant species [9]. Rhizobial and actinorhizal plants evolved nodules, symbiotic organs that form in roots; the host supplies carbon and regulates oxygen, while endosymbiotic bacteria convert gaseous nitrogen into biologically available ammonia and nitrate [10,11]. These diazotrophic symbioses have been extensively studied in terms of the biochemical interactions between microbes and plant cells [12]. Another symbiosis between roots and microbes is the ‘rhizophagy cycle’, where plants secrete exudates into the soil, attracting bacteria that they internalize into root cells at the root tips [13,14,15]. Plants then extract nutrients from the intracellular bacteria using reactive oxygen (superoxide) produced in root cell plasma membranes by NADPH (Nicotinamide Adenine Dinucleotide Phosphate) oxidases [16]. Nutrients extracted oxidatively from internalized microbes supplement nutrients obtained from microbe-mediated solubilization of soil nutrients and from mycorrhizal symbioses [16].

Although roots are the site of the best-understood mutualisms with microbes, the aerial parts of plants represent perhaps the largest biological surface on earth, estimated at about 1,017,260,200 km^2^, and host innumerable microbial cells whose functions are not fully understood [17]. Bacteria are the dominant type of microbe in the phyllosphere, where their growth is limited by access to water, carbon, and nitrogen [18], and their perceived contribution to plant health is mostly limited to instances where they prevent leaf colonization by pathogens [19]. Epiphytic microbes fix significant amounts of nitrogen on leaf surfaces [18,19,20,21,22], and although they have been shown to influence the internal endophytic leaf metabolome, the significance of the nitrogen to plants is unknown [23]. Bacteria that enter plant leaves may be better protected from desiccation, UV, and competition with other microbes and have more direct access to carbon, nutrients, and water [24,25], while the products of their nitrogen fixation may be absorbed by the plant. It has previously been reported that the highest concentrations of bacteria in the phyllosphere occur wherever there are abundant nutrients available from localized leakage, such as from trichomes or sites of injury [18,26], suggesting that bacteria could grow within leaves if they could gain access. In particular, trichomes in many different plant species are well-known targets for invasion by pathogens [27]. Biocontrol fungal endophytes live in the trichomes of cacao trees, and symbiotic cyanobacteria colonize the trichomes of *Azolla* (seedless aquatic ferns) [27], suggesting that trichomes could be the location of bacterial nitrogen fixation in other plant species as well. There are some examples of bacteria inside leaves contributing significant nitrogen to the plant [24,28] and other examples where the foliar application of diazotrophs improves the nutrient status of the plant [29,30]; however, the mechanisms involved and the exact locations of the microbes are unknown [31].

Ample evidence over the past several decades suggests that all plants internalize microbes into their cells and tissues [13,32,33,34,35,36,37,38,39,40]. The obvious benefits to microbes are nutrients and protection when living inside the plant; plants also benefit from atmospheric nitrogen fixed by endophytic microbes [36,37,38,39,40,41,42,43,44,45,46,47,48,49,50,51]. The mechanisms for the transfer of fixed nitrogen from bacteria to plant tissues are largely unknown. Furthermore, it has been reasoned that photosynthesis would raise oxygen in plant tissues to levels that would make nitrogen fixation impossible without specialized organs to limit oxygen (i.e., nodules) around bacterial endosymbionts [39,40,41,42,43,44,45,46].

Macedo-Raygoza et al. [36] demonstrated through isotopic tracking experiments that an intracellular bacterium, *Enterobacter cloacae*, in root cells established a stable nitrogen-transfer symbiosis with Cavendish banana plants. However, the mechanism for this stable nitrogen transfer between bacteria and root cells is not fully understood. More recently, Chang et al. [52] showed evidence of the possible exchange of plant carbon (sugars) for bacterial nitrogen (nitric oxide and nitrate) in root hairs with histochemical experiments on intracellular bacteria in root cells. Here, intracellular bacteria displayed a prolonged interaction with plant root hairs, where they secreted ethylene, triggering host cells to grow and supply exudates, including carbohydrates. Simultaneously, the plant cell produced superoxide, which appeared to induce bacteria to secrete antioxidant nitrogen (nitric oxide or other nitrogenous antioxidants) that may combine with superoxide to produce nitrate. In this case, nitric oxide was thought to be derived from the activity of microbial nitric oxide synthase and be distinct from fixed nitrogen produced within the more efficient nitrogen-transfer symbioses in the nodules of legumes, where ammonia may be secreted by bacteria.

The present study was designed to survey leaf cells for the presence of intracellular bacteria and to evaluate the possible occurrence of nutrient exchanges between bacteria and plant cells via several experiments. Because they are the sites of the highest carbon emission into the phyllosphere [53,54] and the location of the largest aggregates of bacterial cells in the phyllosphere [55,56], we hypothesized that bacteria from the leaf surface, perhaps growing on shoot meristems and early developing leaves [57], would be absorbed into leaves or otherwise find their way into leaf cells (e.g., leaf epidermal cells, trichomes [58,59,60], or other non-photosynthetic cells), where they may be active in sustained chemical exchanges with the plant host.

## 2. Materials and Methods

### 2.1. Plant Materials

Several plants or seeds were obtained from wild populations available in the United States or territories [61] (Table 1). For cultivated species, including *Cannabis sativa*, *Citronella mucronata*, *Festuca rubra*, *Glycine max*, *Hosta plantaginea*, *Humulus lupulus*, *Perilla frutescens*, and *Vigna radiata*, plant tissues or seedlings were obtained from commercial sources. Plants were selected on the basis of leaf structure, i.e., those without trichomes (e.g., *Agave* spp. *Hosta plantaginea* and *Vanilla phaeantha*), those with filamentous trichomes (e.g., grasses and *Ailanthus altissima*), and those with glandular trichomes (e.g., *Cannabis sativa*, *Citronella mucronata, Humulus lupulus,* and *Rhus glabra*). In some of the isotopic nitrogen-tracking experiments (Table 2), plants were grown from seeds or cuttings to develop seedlings or young plantlets for the experiments. Plants for these experiments were: *Agave boldinghiana*, *A. palmeri*, *Festuca rubra*, *Hosta plantaginea*, *Humulus lupulus*, *Thespesia populnea*, and *Vanilla phaeantha*. These species were selected because of their capacity to grow or remain vigorous under conditions in 15N_2_ gas chambers for a seven-day period in the laboratory. For seed sterilization experiments, we used *Rhus glabra* and *Perilla frutescens* because preliminary screens showed that these species could withstand the rigorous seed-surface disinfection protocols needed to remove seed microbes. *Ailanthus altissima* was used for the elevated carbon dioxide experiment because the young growing stems and leaves of this plant were abundantly available and leaves were covered with pitted filamentous trichomes, where lateral pitting and nitrate staining could be readily assessed. Seedlings of *Trifolium pratense* were used in the mCherry bacterial transformation and tracking experiment because preliminary experiments showed that these seeds were readily cleaned of bacteria using rigorous seed sterilization and because seedling leaves were small and thus amenable to microscopic examination. For microbe isolation (Table 3), we selected *Ailanthus altissima*, *Glycine max*, and *Humulus lupulus* because the former two have abundant pitted-filamentous trichomes and the latter possesses abundant glandular trichomes. Other species listed in Table 3 were either tested previously by us or other investigators and are included because the bacteria were isolated and characterized from leaves or bracts of species used in our experiments.

### 2.2. Histochemical Staining of Intracellular Bacteria

Microscopy was carried out using a Zeiss Axioskop compound light microscope under brightfield or phase-contrast conditions. Histochemical staining was performed using the following protocols.

#### 2.2.1. Reducing Sugar Staining

Copper sulfate (1% aqueous) was used to stain for reducing sugars. In the presence of reducing sugars (e.g., glucose or fructose), copper sulfate becomes yellow to orange in color [62].

#### 2.2.2. Ethylene Staining

For ethylene staining, seedlings were stained in 1% ammonium molybdate (Sigma-Aldrich, St. Louis, MO, USA) solution in water for 10–20 min [63]. A blue to purple color in and around bacteria in plant cells was a positive indication of ethylene production. For ethylene detection, we also used 1% potassium permanganate in water [64,65]. This reagent gave a brown color around bacteria where ethylene was produced. In some cases, we used a saturated solution of sulfur monochloride that gave a brown color around bacteria releasing ethylene [66].

#### 2.2.3. Superoxide Staining

To detect superoxide, a 0.1% (*w*/*v*) water solution of nitro blue tetrazolium chloride (NBT) (Sigma-Aldrich) was used [67,68]. Seedling roots were soaked in NBT stain for 30 min at lab ambient temperature prior to examination by light microscopy as described above. Superoxide was visualized as a blue to purple color in association with microbes.

#### 2.2.4. Hydrogen Peroxide Staining

Hydrogen peroxide was visualized around microbes within plant cells through the use of the stain 3,3-diaminobenzidine tetrahydrochloride (DAB) [69,70]. For this, we used buffered tablets of DAB obtained from Sigma-Aldrich, where tablets were dissolved in 10 mL of water. To stain plant tissues, plants were immersed in the DAB solution for 5 to 12 h under lab ambient conditions. Hydrogen peroxide was visualized as a brown color around microbes in plant cells using light microscopy.

#### 2.2.5. Nitric Oxide Staining

We used 0.1% ferric sulfate acidified with sulfuric acid (10 mL of concentrated H_2_SO_4_ in 90 mL of ferric sulfate solution) to visualize nitric oxide around bacteria in plant cells [62]. Staining was accomplished by applying the stain solution onto plant tissues for 10 min, followed by microscopic examination. Nitric oxide was indicated as a brown pigmentation consisting of an iron–nitric oxide complex visualizable around bacteria within cells.

#### 2.2.6. Nitrate Staining

Nitrate was detected around microbes in plant cells using a nitrate stain composed of 0.1% (*w*/*v*) diphenylamine (Sigma-Aldrich) in 20% (*v*/*v*) sulfuric acid [71]. Seedling tissues were stained by putting tissues on a slide with a drop of acidified diphenylamine stain. Nitrate was indicated by a blue or purple color around bacteria in plant cells.

### 2.3. Differential Nitrogen Assimilation Experiments

To conduct differential 15N_2_ assimilation experiments, previous methods were followed [69,70,71,72] (Table 2). Here, root-bearing but soilless plants of *Agave* spp., *Festuca rubra*, *Hosta plantaginea*, and *Thepesia populnea* (with roots wrapped in moist paper towels) or rootless plants of *Ailanthus altissima*, *Humulus lupulus*, *Lonicera japonica*, and *Vanilla phaeantha*, where roots were fully excised from stems (with the cut stem immersed in water), were placed in sealed 11 L chambers and the air enriched with 33 mls of 15N_2_ (Sigma-Aldrich, 98% purity) injected under vacuum. Control plants were treated similarly, but air was not enriched with 15N_2_. Plants were incubated under laboratory ambient temperature under 12 h/12 h alternating light/dark for 7 days, after which plant leaves, inflorescences, stems, and roots, if present, were sampled and analyzed for 15N/14N ratios using Isotope-Ratio Mass Spectroscopy at the Stable Isotope/Soil Biology Laboratory of the University of Georgia in Athens, GA, USA. Data analysis was conducted, where means were represented by 3 or more observations (Table 2).

### 2.4. Bacterial Isolation, Identification, and Characterization

In order to determine the possible identities of some of the leaf and bract bacteria from filamentous trichomes (*Ailanthus altissima* and *Glycine max*) and glandular trichomes (*Humulus lupulus*), we isolated bacteria from leaf washings using sterile water. Here, young leaves or bracts were lightly agitated in 10 mL of sterile water, then approximately 0.5 mL was spread onto Jensen’s nitrogen-free media, and bacteria that grew were selected for further characterization (Table 3). To identify bacteria, 16S rDNA genes of the bacteria were sequenced and used to identify bacteria, as outlined elsewhere [73]. For bacterial identification, ~900 base pairs of the 16S rDNA region were obtained [74]. Table 3 also lists some bacteria isolated in previous studies of leaf-associated bacteria. Specific identifications were made by some authors through additional use of other bacterial genes referenced in Table 3. For new isolates, sequences were compared to sequences available in the NCBI GenBank database to identify the closest matches. Sequences of new isolates were submitted to GenBank (Table 3). For some of the bacteria, acetylene reduction assays (ARA) were conducted to assess possible nitrogen fixation by bacteria (Table 3). Here, pure cultures of isolates were inoculated into nitrogen-deficient semisolid maleate (NDSM) media and incubated at room temperature for 24 h prior to acetylene reduction assay. Nitrogenase activity using ARA was determined using previously described methods [75,76]. In some cases (Table 3), nitrogen fixation capacity was inferred from growth in nitrogen-free media [75]; in other cases, nitrogenase genes were detected in bacteria (Table 3). The data in Table 3 are included to show some of the bacteria that have been isolated from leaves and bracts of plants and are not intended to be an exhaustive study of microbial communities within leaves and bracts or the nitrogenase capacity of microbes.

### 2.5. Experiments to Assess Effects on Trichomes by Reduction in Seedling Bacteria

Several experiments were conducted to determine effects on trichomes when bacteria were reduced or removed through the sterilization of seeds. These are outlined below.

#### 2.5.1. *Rhus glabra* Seed Sterilization Experiment

Following a previously published sterilization protocol [61], seeds of *Rhus glabra* were superficially cleaned of surface tissues and microbes by soaking for 10 min in concentrated sulfuric acid (95%), followed by washing and agitation in 4% sodium hypochlorite for 30 min at laboratory ambient temperature, and then washed in several changes of sterile water. Surface-disinfected seeds and non-disinfected control seeds were planted in sterile vermiculite and incubated under fluorescent lights for two weeks to permit germination and seedling growth. Seedling leaves of treatments and controls were then examined microscopically for the presence of bacteria within glandular trichomes, for nitric oxide using iron sulfate stain, and for nitrate using acidified diphenylamine, as described above. Leaves of seedlings that formed in all experiments were examined microscopically after staining for nitric oxide and nitrate to determine whether trichome development and nitrogen staining were negatively impacted.

#### 2.5.2. *Perilla frutescens* Seed Sterilization Experiment

Following previously published protocols [15,52], seeds of *Perilla frutescens* were surface disinfected by agitation in 4% sodium hypochlorite for 30 min at laboratory ambient temperature before being washed in several changes of sterile water. Surface-disinfected seeds and non-disinfected control seeds were planted in sterile vermiculite and incubated under fluorescent lights. After two weeks, leaves of treatments and controls were examined microscopically for the presence of bacteria within glandular trichomes, nitric oxide, and nitrate, as described above.

#### 2.5.3. Bacterial Replication Repression in *Ailanthus altissima* Seedlings Using Elevated Carbon Dioxide

Previous experiments using bacteria in root hairs have shown that elevated carbon dioxide (CO_2_) suppresses superoxide production and reduces the replication of bacteria in root hairs [52]. To evaluate whether elevated CO_2_ could suppress bacterial replication on trichomes by suppressing superoxide production by plant cells, as previously observed in root hairs [77], seeds of *Ailanthus altissima* were lightly disinfected in 4% sodium hypochlorite for five minutes with continuous agitation to remove superficial seed bacteria before being planted in moist sterile vermiculite in aluminum trays (10 seeds/tray). One tray was placed in an 11 L sealed chamber containing dry ice to elevate carbon dioxide to approximately 180,000 ppm (~0.3 g dry ice/L air). The other tray was placed in an 11 L chamber with air (at approximately 410 ppm carbon dioxide). After 3 weeks, seedlings from both treatments were examined. Dry ice and air were replaced in chambers every week to maintain gas levels in chambers.

### 2.6. Fluorescent Protein mCherry Transformation of Klebsiella oxytoca

To transform *Klebsiella oxytoca* isolated from *Agave palmeri*, we used the plasmid pSEVA237R_Pem7, a self-replicating, broad-host-range plasmid that is not inserted chromosomally and does not contain any transposons [78,79]. pSEVA237R_Pem7 contains an mCherry gene expressed under the constitutive promoter Pem7 and a kanamycin resistance gene, allowing for the continuous production of mCherry under kanamycin selection. *Klebsiella oxytoca* cells were grown overnight in LB broth (Sigma-Aldrich, St. Louis, MO, USA) at 23 °C (OD_600_ = 2.5) and collected via centrifugation at 12,000 rpm for 5 min. The cells were rinsed once in 1 mL of ddH_2_O and then resuspended in 100 μL of ddH_2_O. Then, 500 ng of plasmid DNA was added to 100 μL of cell suspension, and the mixture was transferred to a 2 mm electroporation cuvette (Bio-Rad, Hercules, CA, USA). Cells were electroporated at 2.5 kV/cm, 25 μF, and 200 Ω using a Gene Pulser (Bio-Rad, Hercules, CA, USA) and then immediately resuspended in 1 mL of LB and incubated at 37 °C for 1 hr. After incubation, 100 μL of cell suspension was plated onto LB agar plates amended with 50 μg/mL kanamycin. Pink colonies were cultured further on LB agar plates with kanamycin, and mCherry production was confirmed using a confocal microscope (Zeiss LSM 710; Oberkochen, Germany).

### 2.7. Inoculation Experiments

Bacteria (*Klebsiella oxytoca*) from *Agave palmeri*, labeled using the fluorescent tag mCherry, were used to inoculate axenic (disinfected using 4% NaOCl for 30 min with agitation) seeds of *Trifolium pratense*. Seeds were inoculated by soaking them in a cell suspension (1 loopful of bacteria in 1 mL of sterile DI water) for 10–15 min. Inoculated seeds and non-inoculated seeds were incubated for 1 week on sterile agarose under alternating light conditions at lab ambient temperature. Seedling leaves were then examined using confocal microscopy to assess the entry of bacteria into leaf cells.

### 2.8. Confocal Microscopy

To visualize the entry of mCherry-labeled *Klebsiella oxytoca* into clover leaf cells, inoculated clover seeds were placed into agarose plates amended with 75 μg/mL kanamycin to induce the continued production of mCherry. The seeds were allowed to grow in the agar for 3 days in a 12 h day–night cycle before they were subjected to high levels of CO_2_ (5–6 g of dry ice in 2 L of air) for 24 h to suppress host-produced superoxide around bacteria. This enables bacteria to recover from exposure to superoxide. Leaf tissue samples were additionally stained with Calcofluor White M2R (Sigma-Aldrich, St. Louis, MO, USA) to visualize plant cell walls and SYTO13 (ThermoFisher Invitrogen, Waltham, MA, USA) to visualize nucleic acids in unstained bacteria and plant cells. Then, 405 nm, 488 nm, and 594 nm lasers were used to excite the Calcofluor White M2R, SYTO13, and mCherry stains, respectively.

### 2.9. Statistical Analyses

Statistical analyses were performed for isotopic nitrogen assimilation experiments (Table 2). In Table 2, simple numerical means ± standard deviations are reported. We further conducted t-tests (unequal variance; Excel Data Analysis Toolpak) comparing plant parts of treated (15N_2_) vs. untreated (air) plants, where the number of observations was 3 or more. The *p*-values are reported in Table 2. For *Thespesia populnea* and *Vanilla phaeantha*, we conducted repeated measures analysis of variance (ANOVA) with Tukey’s HSD post hoc comparisons (Vassarstats; alpha = 0.05), where groups with the same letter are not statistically different from each other.

## 3. Results

### 3.1. Histochemical Staining of Intracellular Bacteria

**Figure 1:**
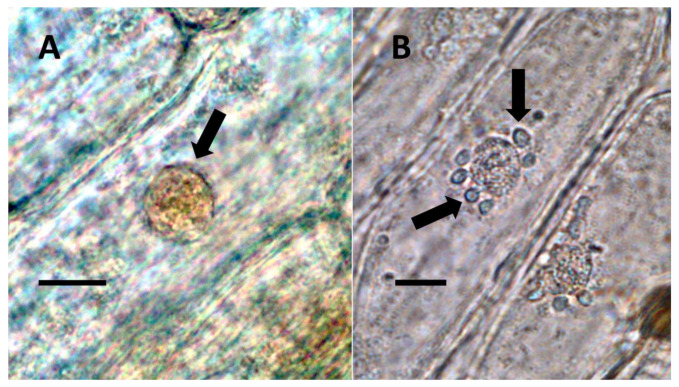
Nuclei showing internal bacteria (arrows). (**A**) *Festuca rubra* nucleus (arrow) stained for reducing sugars (orange color in nucleus) using copper sulfate (Bar = 10 µm). (**B**) *Hosta plantaginea* nuclei stained with acidified diphenylamine to show nitrate (blue-purple color) as bacteria exit the nucleus (Bar = 10 µm).

**Figure 2:**
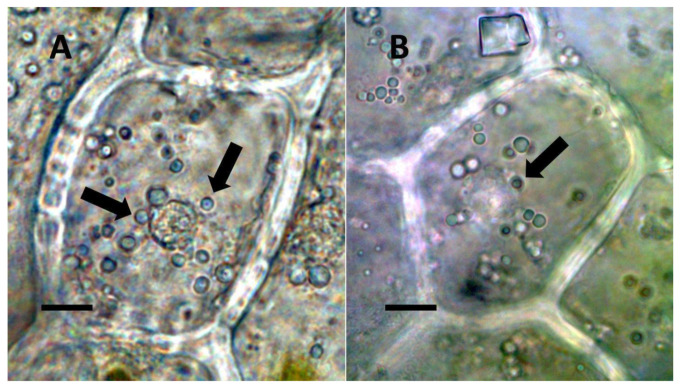
*Vanilla phaeantha* showing bacteria within and emerging from (arrows) nuclei. (**A**) Nucleus stained with acidified diphenylamine to show nitrate (blue-purple color) (Bar = 10 µm). (**B**) Nucleus stained with nitro blue tetrazolium to show superoxide (blue color) around bacteria (arrows) emerging from nucleus into cytoplasm (Bar = 10 µm).

**Figure 3:**
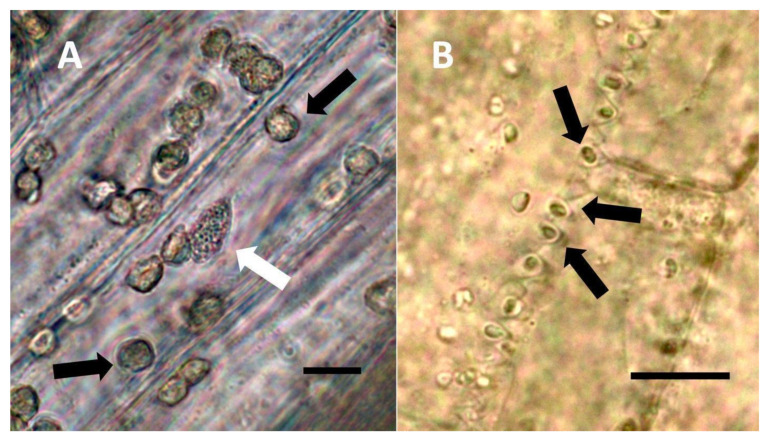
*Festuca rubra* leaf cells showing bacteria within non-photosynthetic cells. (**A**) Leaf sheath mesophyll cells stained with acidified diphenylamine to show nitrate (purple color) around bacterial masses (black arrows) and in nucleus (white arrow) (Bar = 10 µm). (**B**) Developing grass epidermal cells stained with nitro blue tetrazolium to show superoxide (blue color) around bacteria (arrows) accumulating in the serrate lateral walls of the cells (Bar = 10 µm).

**Figure 4:**
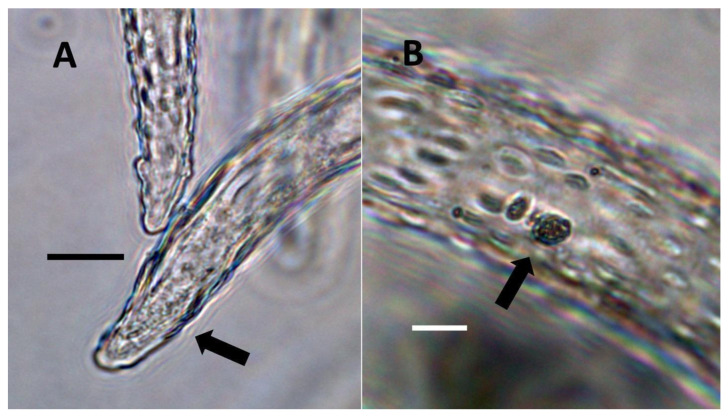
Pitted trichome of *Ailanthus altissima* showing bacteria. (**A**) Developing trichome stained with acidified diphenylamine showing nitrate (blue color) around bacteria (arrow) in the tip of the trichome (Bar = 10 µm). (**B**) Trichome stained with sulfur monochloride to show bacteria (arrow) emerging from lateral pits in wall (Bar = 10 µm).

**Figure 5:**
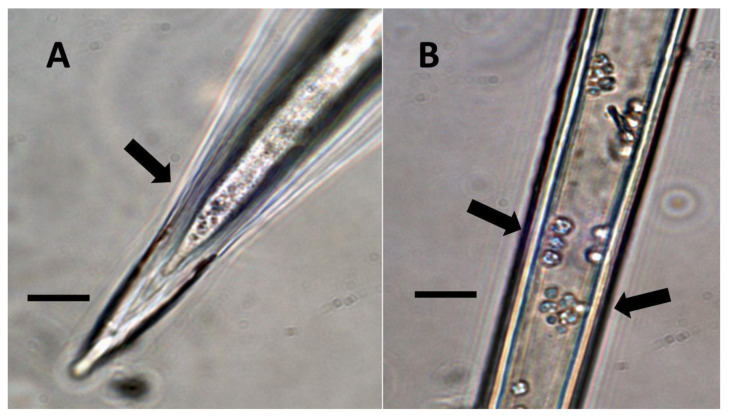
Non-pitted trichomes of *Phragmites australis* showing internal bacteria. (**A**) Ligule trichome stained with iron sulfate to show nitric oxide (brown color) around bacteria (arrow) in hair tip (Bar = 10 µm). (**B**) Ligule trichome stained with ammonium molybdate to show ethylene (blue-purple color) around bacteria (arrows) in hair (Bar = 10 µm).

**Figure 6:**
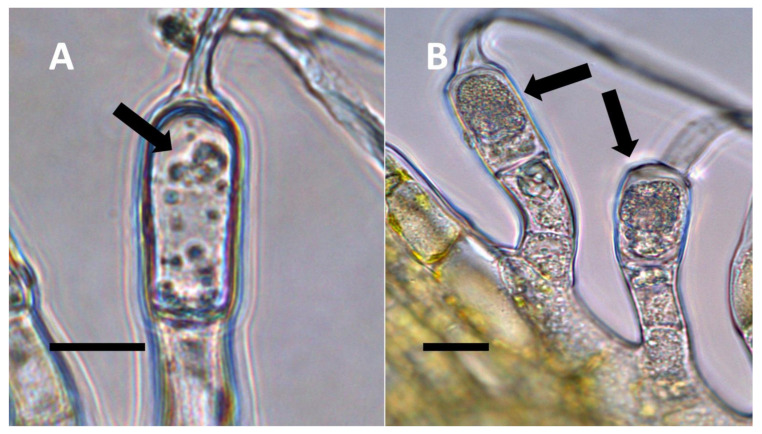
*Solidago canadensis* inflorescence bract non-pitted trichomes. (**A**) Developing trichome stained with acidified diphenylamine showing nitrate (blue color) around bacteria (arrow) in hair (Bar = 10 µm). (**B**) Trichome stained with acidified diphenylamine showing nitrate (blue color) around bacterial masses (arrows) (Bar = 10 µm).

**Figure 7:**
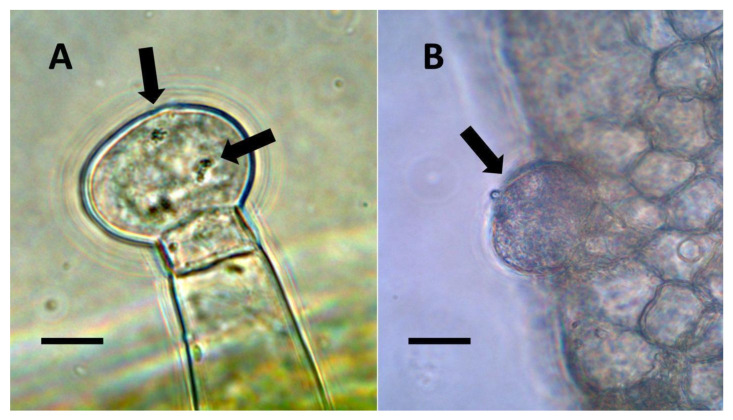
Glandular trichomes of *Verbascum thapsus* and *Humulus lupulus*. (**A**) *Verbascum thapsus* leaf glandular trichome stained with acidified diphenylamine to show nitrate (blue-purple color) around bacteria (arrows) in trichomes (Bar = 10 µm). (**B**) *Humulus lupulus* inflorescence bract glandular trichome (arrow) stained with acidified diphenylamine to show nitrate (blue-purple color) accumulation in trichome (Bar = 10 µm).

**Figure 8:**
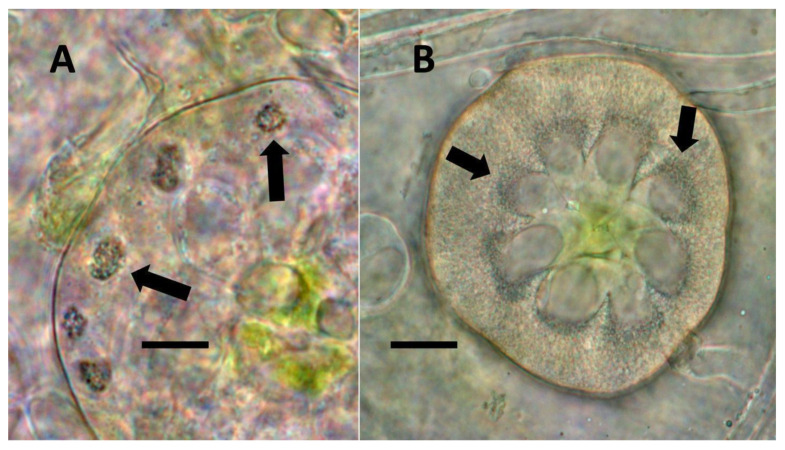
Glandular trichomes of *Cannabis sativa*. (**A**) Developing glandular trichome of hemp stained with nitro blue tetrazolium to show superoxide (blue-purple color) around clusters of bacteria (arrows) in hair (Bar = 10 µm). (**B**) More mature glandular trichome of hemp stained with nitro blue tetrazolium to show superoxide (blue-purple color) (arrows) at the interface of central plant cells and peripheral bacteria in hair (Bar = 10 µm).

**Figure 9:**
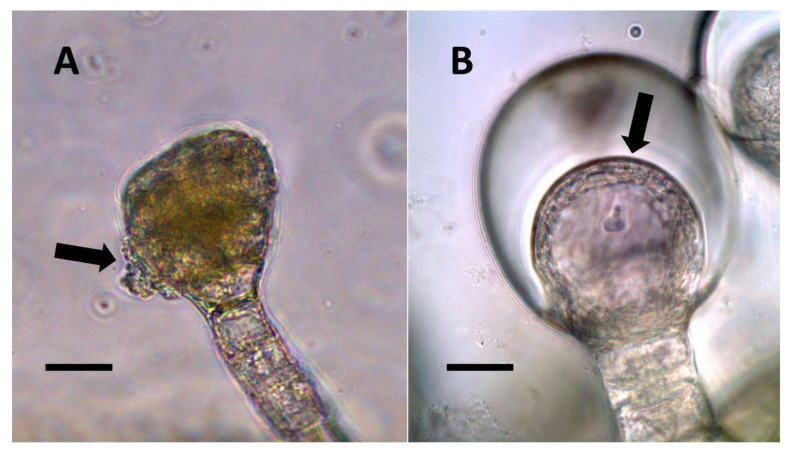
Glandular trichomes of *Lonicera japonica* and *Citronella mucronata*. (**A**) Developing glandular trichome of *Lonicera japonica* stained with iron sulfate to show nitric oxide (brown color) in trichome and bacteria emerging from trichome (arrow) (Bar = 10 µm). (**B**) Glandular trichome of *Citronella mucronata* stained with iron sulfate to show nitric oxide (brown color) (arrow) around bacteria in hair (Bar = 10 µm).

**Figure 10:**
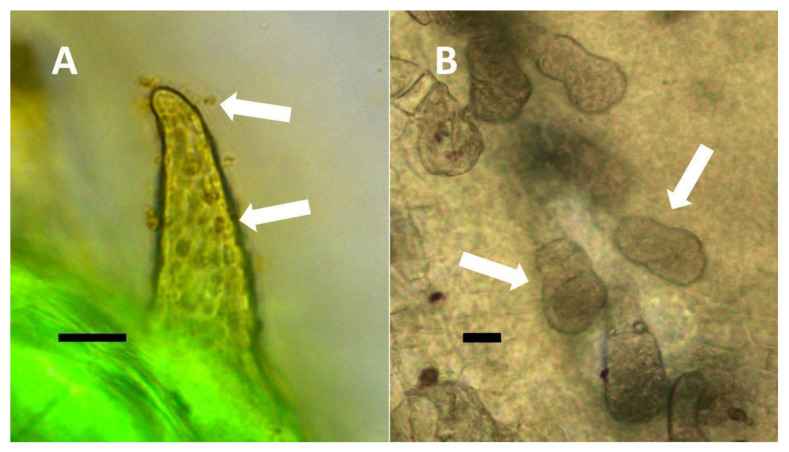
Leaf trichomes of *Solanum dulcamara*. (**A**) Developing pitted filamentous trichome stained with potassium permanganate to show ethylene (brown color) around bacteria (arrows) emerging from and within pits of trichome (arrow) (Bar = 10 µm). (**B**) Glandular trichomes (arrows) along leaf veins stained with acidified diphenylamine to show nitrate (blue-purple color) in hairs (Bar = 10 µm).

**Figure 11:**
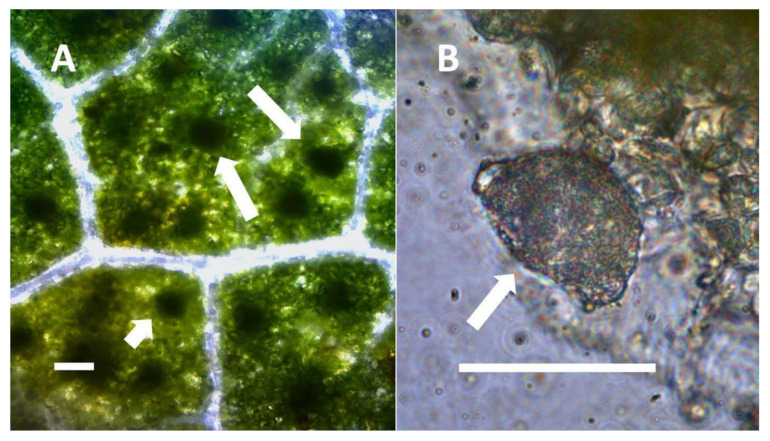
*Thespesia populnea* leaf nodule bacteria. (**A**) Leaf stained with acidified diphenylamine to show bacterial masses (leaf nodules; arrows) (Bar = 50 µm). (**B**) Mass of bacteria (arrow) from leaf nodule stained with acidified diphenylamine to show nitrate (blue color) around bacteria (Bar = 50 µm).

Through histochemical staining and microscopic examination, we found that bacteria were most common in non-photosynthetic cells of leaves and bracts, epidermal and mesophyll parenchyma, and trichomes (filamentous and glandular) (Table 1; Figure 1, Figure 2, Figure 3, Figure 4, Figure 5, Figure 6, Figure 7, Figure 8, Figure 9, Figure 10 and Figure 11). We further found that bacteria could be seen within the nuclei of non-photosynthetic cells of many plants (Table 1; Figure 1, Figure 2, Figure 3, Figure 4, Figure 5, Figure 6, Figure 7, Figure 8, Figure 9, Figure 10 and Figure 11). Through selective histochemical staining, we found evidence for the following chemicals in association with intracellular bacteria: (1) ethylene, (2) reducing sugars, (3) superoxide, (4) hydrogen peroxide, (5) nitric oxide, and (5) nitrate (Table 1; Figure 1, Figure 2, Figure 3, Figure 4, Figure 5, Figure 6, Figure 7, Figure 8, Figure 9, Figure 10 and Figure 11).

### 3.2. Summary of Types of Endosymbiosis in Cells of Leaves and Bracts

We propose to categorize endosymbiosis in leaves and bracts into five categories as follows:

(1) Nuclear symbiosis (Figure 1, Figure 2 and Figure 3): In this symbiosis, bacteria are cultivated within nuclei, where sugars may fuel bacterial replication and metabolic activities. The bacteria are released into the cytoplasm of the cell in vesicles. Bacteria, once released from nuclei, begin to secrete ethylene. Bacteria in the cytoplasm are exposed to host-produced superoxide. Bacteria were also seen to stain for nitric oxide and nitrate, perhaps as an antioxidant in response to host-cell-produced superoxide. Nuclear symbioses were seen in plants without trichomes, including *Agave*, *Hosta*, and *Vanilla*. Nuclear symbioses were also seen in the grasses *Phragmites australis*, *Digitaria sanguinalis*, and *Festuca rubra* along with simple non-pitted trichomes. These trichomes are filaments, typically unicellular, and frequently contain bacteria. Grasses show additional epidermal cell modifications, where the lateral walls of cells develop serrations or convolutions. Bacteria in developing epidermal cells accumulate in the wall serrations (Figure 3). Some trichomes produced in the Asteraceae (e.g., *Eupatorium*, *Helianthus*, and *Solidago*) on bracts show evidence of nuclear symbiosis in cells of the trichome. Typically, the trichomes bearing nuclear symbiosis are thick-walled with striations but do not have lateral pits. Previous experiments with vanilla orchids [57] that possess nuclear symbiosis suggest that the epidermal cells become colonized by bacteria in the shoot meristem, where biofilms of bacteria are cultivated, or in the recently differentiated leaves.
(2) Pitted filamentous trichome symbiosis (Figure 4): In this symbiosis, bacteria are seen to be replicated within trichomes, where they are moved through periplasmic streaming, or cyclosis, within hairs to accumulate in equidistantly spaced depressions on the surface of the trichome plasma membrane. The pores develop in the lateral trichome walls just over the bacterial clusters in the trichome plasma membrane depressions. These trichomes often show reducing sugars around bacteria throughout the trichome. Ethylene, nitrogenous compounds, and superoxide can be seen around bacteria, especially associated with lateral wall pits. These bacteria are often seen to spill from hairs through the pits in the trichome walls; this is especially evident in the basal parts of the trichome. These pitted trichomes may also function to populate the plant surface (phyllosphere) with bacteria. Pitted trichomes were observed in many different dicotyledonous plants (Table 1), including, for example, *Celtis occidentalis* and *Eutrochium maculatum*, but were predominant in *Ailanthus altissima*, where a *Bacillus* sp. was isolated from leaf washings of young plants. In addition, endospores could be observed on and within trichomes.
(3) Non-pitted filamentous trichome symbiosis (Figure 5 and Figure 6): These trichomes often contain bacteria that are evident in the tips of hairs. Nitrogenous chemicals were evident around bacteria in histochemical experiments. Ligule trichomes observed in *Phragmites australis* and leaf sheath trichomes from the grass *Digitaria sanguinalis* appear to be this type. Another example is the highly branched trichomes observed covering heavily tomentose leaves of *Verbascum thapsus*. Bacteria do not appear to exit trichomes in this endosymbiosis. Peltate trichomes observed in *Thespesia populnea* and other species are a special case of this type, where multiple filaments fuse to form a circular sheet, with bacteria present in the tips of each cell of the trichome. Some trichomes of this non-pitted filamentous type do not show evidence of bacteria within them. An example here is *Stachys byzantina*, where leaves are covered with very long filamentous trichomes that do not contain bacteria (Table 1).
(4) Glandular trichome symbiosis (Figure 7, Figure 8, Figure 9 and Figure 10): Some of the dicotyledonous plants examined possessed glandular trichomes that contained bacteria (Table 1). Typically, the tip or head of the glandular trichomes contained several non-photosynthetic plant cells in addition to bacteria. Glandular trichomes tended to stain densely for nitrate compared to other trichome types, suggesting that they are more efficient than other trichomes in nitrogen acquisition. Glandular trichomes with bacteria were notable in *Cannabis sativa*, *Citronella mucronata*, *Humulus lupulus*, *Perilla frutescens*, *Rhus glabra*, *Solanum dulcamara*, *Apocynum cannabinum*, and *Solanum lycopersicum*.
(5) Leaf nodule symbiosis (Figure 11): We observed large masses of regularly spaced bacteria in leaves of *Thespesia populnea* (family Malvaceae). These masses were found to stain densely for nitrate using acidified diphenylamine, suggesting that they were active in producing nitrogen. These structures in leaves correspond to previously described structures called ‘leaf nodules’ [80]. We often observed abundant trichomes with bacteria in developing leaves of *Thespesia populnea*. This emphasizes that plants often show multiple types of structures that may produce nitrogen in their tissues.

### 3.3. Isotopic Nitrogen Assimilation Experiments

In order to evaluate whether nitrogen may be assimilated into tissues of plants containing microbes, we conducted isotopic nitrogen (15N_2_) assimilation experiments. Here, we found that the plants tested showed the highest nitrogen assimilation into leaves or inflorescence bracts and lower levels of assimilation into roots, with the tendency to show the highest levels of absorption into developing leaves (Table 2).

### 3.4. Summary Data on Leaf Bacteria

Several species of bacteria were isolated from leaf tissues in this study and previous studies (Table 3).

**Table 3:** Bacteria isolated from plants with leaf cell endosymbioses.

Host	Organs	Bacterium	GenBank Accession	Growth on N-Free Media	Acetylene Reduction Assay	Nif Genes Assessed	Article
*Agave palmeri*	Seeds, leaves, roots	*Klebsiella oxytoca*	KJ667735.1	**+** ^1^	**+**	N/A	[69]
*Ailanthus altissima*	Leaves	*Bacillus* sp.	OM223869	**+**	N/A	N/A	This article
*Digitaria ischaemum*	Seeds, leaves, roots	*Pantoea* sp.	MK733357	**+**	N/A	N/A	[81]
		*Staphylococcus* sp.	MT275650.1	**+**	N/A	N/A	[81]
*Glycine max*	Leaves	*Bacillus megaterium*	OL870610	**+**	N/A	N/A	This article
*Hedera helix*	Seeds, leaves, roots	*Bacillus amyloliquefaciens*	KM822602	**+**	**+**	**+**	[82]
*Hosta plantaginea*	Seeds, leaves,	*Bacillus amyloliquefaciens*	KM454171	**+**	**+**	**+**	[83]
	Seeds, leaves	*Curtobacterium* sp.	-	**+**	N/A	N/A	[83]
*Humulus lupulus*	Inflorescence bracts	*Pseudomonas fluorescens*	GCA004794015	N/A	N/A	**+** ^2^	[84]
	Inflorescence bracts	*Pseudomonas stutzeri*	GCA_004793985	N/A	N/A	**+** ^2^	[84]
	Inflorescence bracts	*Massilia* sp.	OM223867	**+**	N/A	N/A	This article
	Inflorescence bracts	*Pantoea* sp.	OM223868	**+**	N/A	N/A	This article
*Phragmites australis*	Tillers, leaves	*Bacillus amyloliquefaciens*	KP860304.1	**+**	**+**	**+**	[44]
	Tillers, leaves	*Microbacterium oxydans*	KP860310.1	**+**	**+**	N/A	[44]
	Tillers, leaves	*Achromobacter spanius*	KP860309.1	**+**	**+**	N/A	[44]
*Thespesia populnea*	Seeds, leaves	*Bacillus amyloliquefaciens*	KX622564	**+**	N/A	N/A	[61]
	Seeds, leaves, roots	*Pseudomonas oryzihabitans*	KY471285	**+**	N/A	N/A	[61]
*Vanilla phaeantha*	Leaves, roots	*Bacillus amyloliquefaciens*	KF765481	**+**	N/A	N/A	[57]

^1^**+** = positive result in test; N/A = not assessed. ^2^ Entire genome sequenced and nif genes found in annotations in Pathosystems Resource Integration Center (PATRIC) database.

### 3.5. Experiments to Assess Effects on Trichomes by Reduction in Seedling Bacteria

#### 3.5.1. *Rhus glabra* Seed Sterilization Experiment

**Figure 12:**
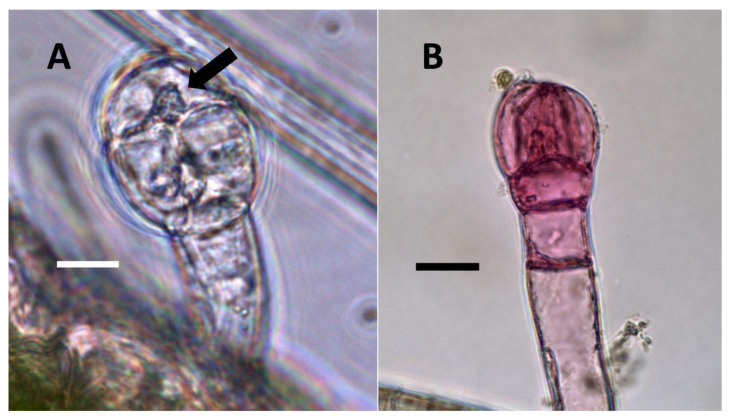
*Rhus glabra* seedling leaf trichomes stained for nitric oxide using iron sulfate. (**A**) Non-sterilized seedling trichome stained with iron sulfate to show nitric oxide (brown color) around bacteria (arrow) internally (Bar = 10 µm). (**B**) Trichome on seedling developed from surface-sterilized seeds stained with iron sulfate showing smaller size and absence of nitric oxide staining internally. The pink color may be due to anthocyanins or other pigments (Bar = 10 µm).

Seedlings that developed from rigorously sterilized seeds showed trichomes that were poorly formed without bacterial contents. Further, trichomes in these seedlings did not stain for nitric oxide internally (Figure 12).

#### 3.5.2. *Perilla frutescens* Seed Sterilization Experiment

**Figure 13:**
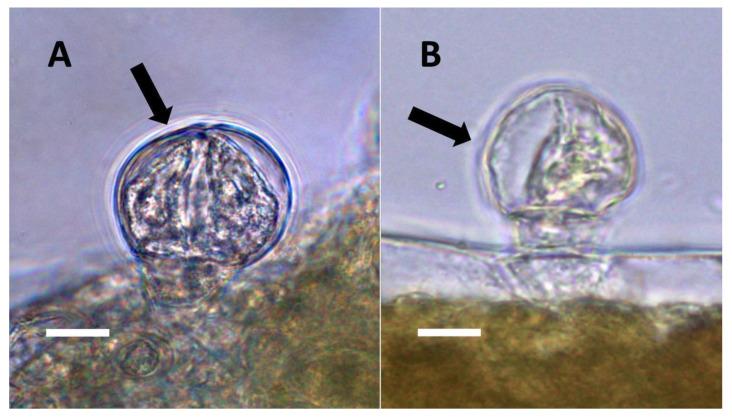
*Perilla frutescens* seedling leaf glandular trichomes (arrows) stained for nitrate. (**A**) Non-sterilized seedling glandular trichome (arrow) stained with acidified diphenylamine to show nitrate (blue-purple color) around bacteria (Bar = 10 µm). (**B**) Glandular trichome on seedling developed from surface-sterilized seeds, stained with acidified diphenylamine showing smaller size and absence of nitrate staining internally (Bar = 10 µm).

Surface-disinfected seeds (>50) produced seedlings that showed reduced stature (size) compared to control seedlings derived from seeds (>50) that were not surface-disinfected (data not shown). In seedlings grown from surface-disinfected seeds, we found that glandular trichomes on leaves of the seedlings showed the absence of bacteria internally and a failure to stain for nitric oxide and nitrate (Figure 13).

#### 3.5.3. Bacterial Replication Repression in *Ailanthus altissima* Seedlings Using Elevated Carbon Dioxide

**Figure 14:**
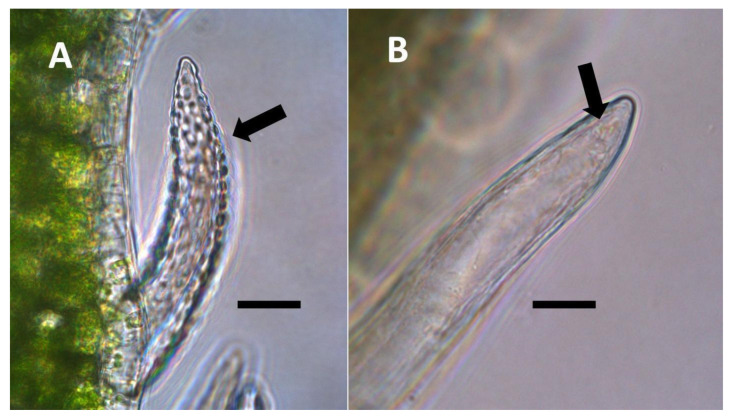
Trichomes of *Ailanthus altissima*. (**A**) Pitted filamentous trichome (arrow) grown in air, stained with acidified diphenylamine to show nitrate (blue-purple color) in hair and abundance of lateral pits (Bar = 10 µm). (**B**) Trichome grown in air with elevated carbon dioxide, stained with acidified diphenylamine showing absence of nitrate and lateral pits; non-replicated bacterial rods (arrow) are seen in the hair tip (Bar = 10 µm).

In experiments using elevated carbon dioxide to suppress superoxide formation in trichomes and bacterial replication, we saw that trichomes formed on seedling leaves in elevated carbon dioxide frequently showed few bacteria internally, the absence of nitrate staining, and the absence of pits in lateral walls of the trichome (Figure 14).

#### 3.5.4. Inoculation Experiments

**Figure 15:**
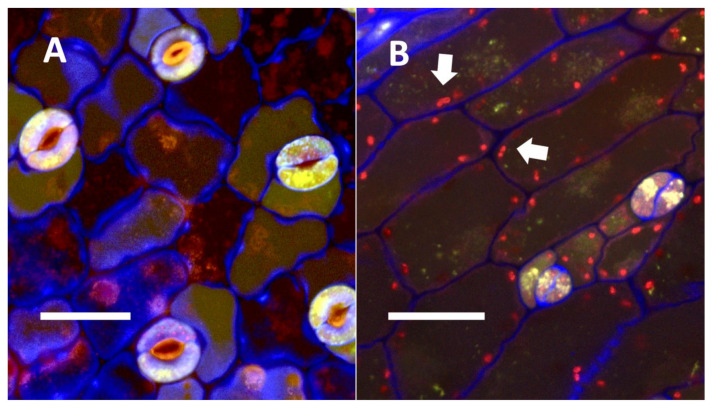
Confocal image of seedling leaves with and without mCherry-tagged bacteria (*Klebsiella oxytoca*) within epidermal cells. (**A**) Uninoculated control of *Trifolium pratense* leaf from inoculation experiment without mCherry-tagged bacteria within leaf epidermal cells (Bar = 10 µm). (**B**) Inoculated leaf of *Trifolium pratense* from inoculation experiment with red mCherry-tagged bacteria (arrows) within leaf epidermal cells (Bar = 10 µm).

**Figure 16:**
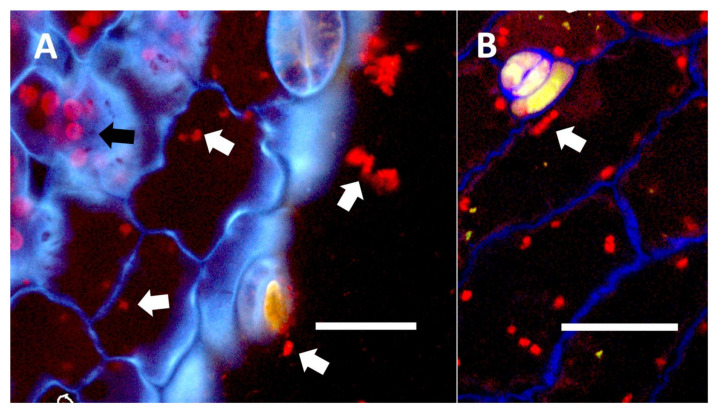
*Trifolium pratense* seedling leaves showing bacteria within epidermal cells. (**A**) *Trifolium pratense* leaf from inoculation experiment showing red colored mCherry-tagged *Klebsiella oxytoca* cells (white arrows) within epidermal cells and outside the leaf (Bar = 10 µm). Chloroplasts (black arrow) are evident in the mesophyll cells beneath the epidermis. (**B**) *Trifolium pratense* leaf from inoculation experiment showing red colored mCherry-tagged *Klebsiella oxytoca* cells within epidermal cells (Bar = 10 µm). The white arrow shows a chain of bacterial cells.

In inoculation experiments to assess the entry of bacteria into leaf cells using mCherry-tagged bacteria (*Klebsiella oxytoca*) from *Agave palmeri*, we found that mCherry-tagged bacteria inoculated onto seedlings of *Trifolium pratense* colonized in leaf epidermal cells internally (see Figure 15A,B and Figure 16A,B). Bacteria were observed to localize primarily at the lateral margins of epidermal cells and were not seen to colonize the mesophyll cells beneath the epidermis (Figure 15 and Figure 16). This is consistent with previous leaf colonization experiments where bacteria appeared to enter cells at cell margins on meristematic and expanding leaves [57]. Bacterial cells were also seen to divide within cells and sometimes formed long chains in cells (Figure 16B). This is typical behavior for bacterial protoplasts, also termed ‘L-forms’ [52,57]. L-forms are generated in root cells as well in response to superoxide, which may oxidize the walls of bacteria [52]. mCherry-stained bacteria were not observed within leaf epidermal cells of *Trifolium pratense* that were not inoculated (Figure 15A).

## 4. Discussion

Our experiments using histochemical stains (Table 1; Figure 1, Figure 2, Figure 3, Figure 4, Figure 5, Figure 6, Figure 7, Figure 8, Figure 9, Figure 10, Figure 11, Figure 12, Figure 13 and Figure 14) and differential isotopic assimilation experiments (Table 2) suggest that many vascular plants contain bacteria on and within rapidly growing non-photosynthetic cells (e.g., epidermal, subepidermal parenchyma cells, and trichomes) of their leaves and bracts that could be involved in nutrient exchange with plant cells. The nutrients that could be transferred in these endosymbioses include nitrogen that may be fixed from the atmosphere or obtained from other sources before microbes enter plant cells or, alternatively, could be fixed within plant cells (e.g., epidermal cells and trichomes). More work is needed to fully resolve the question of where nitrogenous chemicals detected around bacteria are actually fixed. As reported recently [38], many of these bacteria (including *Pantoea*, *Pseudomonas*, and *Klebsiella*) appear to be taxonomically similar to a pan-angiosperm seed-transmitted microbiome with an otherwise poorly understood function within the plant. These bacteria are able to become internalized at meristems when leaf cells are forming. It has also been shown that some bacteria may be absorbed from soils and transported to leaves [85]. We observed that these trichome openings also serve as exit points for endophytes to colonize the leaf surface, providing a mechanism that helps to explain the provenance of some phyllosphere microbes, including many seed-derived proteobacteria [38]. Glandular trichomes also frequently rupture, which releases microbes to the plant surface. Until now, phyllosphere microbes were primarily thought to derive from environmental sources (bioaerosols and rainfall) or surface contact with animals, seed tissue, the spermosphere, buds, other leaves, and twigs [86]. Once inside the cell, many of these bacteria enter the nucleus (in the case of epidermal cells) or move around the cytoplasm and gather under pits (in the case of pitted filamentous trichomes), where they could be transferring nutrients to plant cells. Plants that were stripped of these seed-transmitted endophytes (or where bacterial replication was suppressed by high carbon dioxide concentrations) had poorly developed trichomes that lacked nitrogen compound accumulation, reinforcing the importance of seed-transmitted microbiomes [38].

We showed evidence that nitrogen is produced around bacteria inside plant cells through histochemical staining for nitric oxide and nitrate. However, because we did not directly detect nitrogenase activity in bacteria, we cannot be certain that nitrogenous chemicals were derived from nitrogen fixation within plant cells. To fuel bacterial growth and perhaps nitrogen fixation, plants would need to provide intracellular bacteria with fixed carbon. Our histochemical staining using copper sulfate showed reducing sugars (likely fructose and glucose, breakdown products of sucrose) around bacteria within plant cells (Table 1; Figure 1). This observation is consistent with the ‘nutrient trap mechanism’ described by Chang et al. [52] in the root hairs of plants. In this suggested mechanism, microbes secrete ethylene, triggering plant cells to release carbohydrates and produce superoxide, with bacteria responding by secreting nitrogenous antioxidants, such as nitric oxide, that may be further oxidized by superoxide to form nitrate [52]. In previous histochemical experiments [52], we confirmed that ethylene was produced around bacteria by using ammonium molybdate stain. In the present study, we added the ethylene stains potassium permanganate and sulfur monochloride to increase support for ethylene detection. In the previous study [52], we also used an inhibitor of microbial ethylene synthase, the arginine analogue *N*_ω_-Nitro-L-arginine methyl ester hydrochloride (L-NAME), to confirm the production of ethylene by bacteria in plant cells. In the presence of L-NAME, no ethylene was evident around bacteria in plant cells due to competitive inhibition of ethylene synthase. In all plant species examined (Table 1), we found histochemical staining evidence for the production of ethylene by endophytic bacteria (Figure 5 and Figure 10) and superoxide by host cells (Figure 2 and Figure 5). Superoxide was detected using nitro blue tetrazolium, which is commonly used to detect superoxide [52]. We further found that nitric oxide and/or its oxidized product (nitrate) were present around bacteria and often accumulated in plant cells so that the entire trichome cell stained densely for nitrate (Figure 7, Figure 9, Figure 10, Figure 13 and Figure 14). In previous experiments [52] for the detection of nitric oxide around microbes in roots, we employed the fluorescent indicator 4,5-diaminofluorescein (DAF) and the nitric oxide inhibitor methylene blue [52]. We also used ferric sulfate acidified with sulfuric acid to visualize nitric oxide around bacteria in root hairs. In the present study, we used only the acidified ferric sulfate stain to detect nitric oxide. We did not use methylene blue when examining trichomes and other leaf and bract cells because trichomes are not present in an aqueous matrix, like root hairs are, where the inhibitor can diffuse freely into plant cells. We detected nitrate using an acidified diphenylamine stain that is commonly employed for nitrate detection [52]. These histochemical experiments do not confirm beyond doubt that the bacteria are the source of the chemicals detected around intracellular microbes, but they are consistent with that hypothesis based on the prior study of bacteria within root hairs [52].

### 4.1. Patterns of Nitrogen Assimilation into Leaves and Bracts

It is possible that the absorption of 15N into tissues is not an accurate measure of the nitrogen benefit from intracellular bacteria. Plants may contain diazotrophic bacteria in many different tissues and organs (not just leaves); however, it was only where we observed bacteria located within plant cells that we saw the production of nitrogenous compounds based on histochemical staining [52]. Differences in 15N assimilation between experiments and plants (Table 2) could relate to the efficiency, abundance, and/or growth rates of endophytic bacteria inside different plant species. Nevertheless, we found that *Thespesia populnea* and *Agave boldinghiana* showed similar patterns of nitrogen absorption (Table 2). These plants appeared to show a gradual increase in nitrogen assimilation into leaves and then a decrease as leaves aged; however, our experiment lacked sufficient replication for statistical certainty. This possible increase may be due to the proliferation of bacteria and possible nitrogen fixation within or on the surface of leaf tissues; the youngest tissues show the highest density of plant cells up to a maximum, fueled by host sugars in growing tissues. Later, as plant tissues age, bacterial replication and nitrogen assimilation appears to decrease, presumably due to a lower supply of sugars from the plant host and the cessation of bacterial replication. Older leaves tended to have fewer bacteria within their nuclei, suggesting that it was lower bacterial replication leading to lower nitrogen accumulation in older leaf cells. A distinct pattern of nitrogen absorption was seen in *Vanilla phaeantha* leaves (Table 2); here, the earliest leaves showed the highest nitrogen absorption, which then gradually waned as leaves aged. This difference between vanilla plants and the others could be due to the peculiar way that *V. phaeantha* cultivates bacterial biofilms in its ‘vase-like’ shoot-tip meristem [57], resulting in the highest bacterial infection occurring in epidermal cell nuclei of the youngest meristematic leaves. The other plants do not have comparable vase-like shoot meristems, although in those plants, it seems likely that leaf infection also begins at the shoot meristems and recently formed organs, furnishing microbes with the shortest route into leaf and bract tissues [57].

### 4.2. Symbiosis Stacking

It is possible that one strategy of plants (such as the dicots hops, tree-of-heaven, and Portia tree) is to employ multiple endosymbioses in various parts of plants, with each perhaps yielding only small amounts of nutrients. This may be referred to as ‘symbiosis stacking’. Such plants may be found, for example, to combine epidermal nuclear endosymbiosis, various types of trichome endosymbioses, and leaf nodules. The clearest evidence of symbiosis stacking was in *Thespesia populnea*, which seems to show moderate levels of isotopic nitrogen assimilation into its leaves (Table 2) and grows in very infertile sandy soils. In the leaves of this tree, we observed nuclear endosymbiosis, non-pitted peltate trichomes, and leaf nodules (Figure 11), while bacterial endosymbiosis is also known to occur within its roots [52]. Many grasses have highly branched root systems that are engaged in rhizophagy [15], as well as endosymbiosis in leaves [52]. Such root symbiosis, when combined with endosymbioses in leaves, may help explain how these monocots can robustly colonize rocky terrain possessing very little soil or nutrient-poor substrates composed largely of sand. Weedy and invasive grasses, such as *Phragmites australis*, have also been observed by us to possess nuclear symbiosis and simple non-pitted trichomes, which, when combined with their extensive and highly efficient root systems, might help explain why these plants can so successfully and aggressively colonize non-native habitats. It is also possible that plants with these endosymbioses may switch to absorb soil nitrogen when that is available. This may be evident in the case of invasive reed grass where plants thrive in nitrogen-contaminated soils [85,86,87,88,89]. More research into stacked symbiosis as a competitiveness and aggressiveness-enhancing trait will need to be conducted to help validate this hypothesis.

### 4.3. Trichome Endosymbiosis

Several lines of evidence support that trichomes host endosymbiotic bacteria that may be functioning in plants to provide nutrients. First, our nitrogen assimilation experiments using *Ailanthus altissima* (Table 2) showed that leaflets bearing abundant trichomes absorbed higher levels of 15N_2_ on an equal weight basis than did the leaf rachis, which was free of trichomes and showed reduced 15N_2_ assimilation. Secondly, when we removed or reduced bacteria in our seed disinfection and elevated CO_2_ experiments, we found that trichomes no longer stained for nitrate (Figure 12, Figure 13 and Figure 14). Kusstatscher et al. [90] found that tomato leaf trichomes are “hot-spots” for alpha-Proteobacteria (many of which are diazotrophic). Blaskovich et al. [91] showed that cannabinoids in *Cannabis sativa* trichomes may inhibit some bacteria, but many Gram-negative potential nitrogen fixers were not inhibited. Multi-omic studies [92] have further shown that trichomes are rich sources of flavonoids, phenols, and enzymatic antioxidants, which might be molecular evidence of the oxidative interactions occurring between trichome cells and diazotrophic bacteria. It has further been shown that the application of bacteria to *Cannabis sativa* plants increased the nitrogen supply and terpenoid content, while the application of inorganic nitrogen reduced the terpenoid content [93]. In another connection between trichomes and nitrogen fixation capacity, Ooki et al. [94] found that the plant symbiosis-related mutation Lot1 (ethylene insensitivity) reduced rhizobial root nodule formation and normal trichome development in the legume *Lotus japonicus*. Nitrogen-fixing plants depend on ethylene as a key hormone regulating the formation of root nodules [95]. Our histochemical experiments (Table 1) suggest the involvement of ethylene in interactions within trichomes, which may likewise affect trichome development, although not to the extent seen in roots, where removal of bacteria results in the complete failure of root hair elongation [52]. Iskra et al. [96] showed that nitrogen fertilization of *Humulus lupulus* resulted in reduced secondary metabolite formation in trichomes of inflorescences. Tang et al. [97] showed that nitrogen-use efficiency in *Cannabis sativa* is reduced when plants are treated with exogenous nitrogen. This makes sense if high nitrogen-use efficiency is related to elevated nitrogen accumulation in *Cannabis sativa* plants due to endosymbiosis, where the application of chemical nitrogen inhibits nitrogen fixation in the bacteria. This is consistent with other studies that show that when chemical nitrogen is applied as a fertilizer, there is a reduction in symbiosis with diazotrophic endophytes and lower rates of symbiotic biological nitrogen fixation [2,37,45,47,49,77,98].

### 4.4. Factors That May Affect Nitrogen Accumulation in Plant Cells

#### 4.4.1. Photosynthate

De-Polli, Boyer, and Neyra [99] used acetylene reduction assays to show that nitrogen absorption by endophytic bacteria in corn tissues was diurnal, increasing during the daylight and subsiding at night. This is logical since sugar is needed to fuel bacterial cell growth and nitrogenase activity, and plant production of sugar is linked to photosynthesis. We observed reducing sugars (Figure 1A; Table 1), likely glucose or fructose from the degradation of sucrose, accumulating around bacteria in the nuclei and trichomes of many plant species (Table 1; Figure 1). Thus, as higher availability of sugars to bacteria in leaves could fuel nitrogenase activity, diazotroph proximity to cells containing chloroplasts could facilitate nitrogen fixation.

#### 4.4.2. Oxygen Levels

De-Polli, Boyer, and Neyra [99] found that the elevation of the oxygen levels in corn tissues reduced nitrogen fixation by bacteria in those tissues. This association occurs because nitrogenase activity is suppressed by high levels of oxygen [96], which is why efficient nitrogen-fixing plants (i.e., nodule formers) reduce oxygen levels around their diazotrophic endosymbionts, using leghemoglobin to bind molecular oxygen [3]. In the nuclear endosymbiosis that we observed in the leaves of monocots and some dicots, bacteria were cultivated within the nuclei surrounded by the nuclear envelope, which may restrict the entry of oxygen into the nuclei of non-photosynthetic epidermal cells (Table 1; Figure 1 and Figure 2). Nuclei are furthermore known to contain nitric oxide and other antioxidants that may reduce oxygen levels to prevent damage to chromosomes; the reduced oxygen in nuclei could also create improved conditions for nitrogenase activity [100,101]. We have histochemical evidence that nitrogenous compounds in bacteria inside the nuclei of plant cells may be harvested after being released from the nucleus into the cytoplasm, where bacteria are subjected to superoxide (Figure 1B and Figure 2A). Thus, there may be distinct periods or locations for bacterial build-up through replication and nitrogen harvesting from bacteria in plant tissues [52]. The formation of trichomes is another way that plants may reduce oxygen around intracellular bacteria, raising them up on stipes that elevate the microbes away from the leaf tissues, where oxygen levels may be high due to photosynthesis.

#### 4.4.3. Variability in Nitrogen Absorption in Plants

Wani, Dart, and Upadhyaya [31] showed that tissues of the leaves, stems, and roots of sorghum and millet may show internal nitrogen fixation, as evidenced by acetylene reduction experiments; however, disturbing or moving plants (especially roots) reduced the rate of nitrogen fixation. This linkage between plant disturbance and reduced nitrogen fixation may be in part the result of the dependency on cell/tissue growth, where abundant carbohydrates may be available or periplasmic streaming may occur, resulting in increased sugars or reduced exposure to reactive oxygen species. Periplasmic streaming is an intracellular microbe cell movement phenomenon where bacteria in the periplasm are seen to move around the cell periphery in the space between the plant cell wall and plasma membrane at a rate of 8–11 µm/s [77]. Verchot-Lubicz and Goldstein [102] hypothesized that plant cell streaming in the cytoplasm plays a critical role in cell mixing. Periplasmic streaming is distinct from cytoplasmic streaming since, at times, there is no obvious connection between the plant cell’s cytoskeleton and the bacteria present in the periplasm. The mechanism for periplasmic streaming is not yet understood. It is also uncertain that bacteria are exclusively in the periplasmic space, and in some cases, bacteria in cells may move into the cytoplasm of the cell, and it is possible that bacteria could be somehow anchored to the cytoskeleton of the plant cell. Tissue disruption may temporarily damage the plant cell’s cytoskeleton and inhibit periplasmic and cytoplasmic streaming and tissue growth [103]. Some of the plants that we tested were minimally disturbed and in the active growth phase; these included *Festuca*, *Hosta*, and *Thespesia* seedlings and the leaves of *Ailanthus* and *Humulus* (Table 2), and consequently, isotopic nitrogen assimilation was notable. Meanwhile, other plants may have been more disturbed during extraction from soils and placement in gas chambers (for example, *Phragmites australis*, *V. phaeantha, Hedera helix*, and *Lonicera japonica*), causing reduced periplasmic streaming and reducing nitrogen assimilation into plant tissues.

### 4.5. Bacteria in Phyllospheres of Plants

Trichome bacteria may be released to the surfaces of plants through lateral pits or through the rupture of the cell wall in the case of glandular trichomes, suggesting that plants can seed their own phyllospheres with diazotrophs—bacteria that are in fact frequently found on leaf surfaces (Table 3) [20,104]. Some of the bacteria that were isolated from leaves of different plant species are listed in Table 3. The genera that we found associated with leaves included *Bacillus*, *Klebsiella*, *Pantoea*, *Pseudomonas*, etc., and any of these could have originated from within trichomes, which in turn may have ultimately originated from the seeds used in our experiments [38]. Further, we observed filamentous pitted trichomes of *Ailanthus altissima* to contain endospores within and on their surface near exit pits, suggesting them as a source of the leaf wash isolates of *Bacillus* sp. (Table 3). In previous experiments involving *Bacillus amyloliquefaciens* from *V. phaeantha*, we found that the bacterium internally colonized seedling leaf cells and even nuclei of *Amaranthus caudatus*, as well as forming endospores in epidermal cells of *V. phaeantha* [57]. In inoculation experiments using an endophytic bacterium (*Bacillus amyloliquefaciens*) from *Hedera helix*, we showed that leaf cells in axenic seedlings of *H. helix* could be recolonized by the microbe [105]. In the present study, we labeled *Klebsiella oxytoca* isolated from *Agave palmeri* with the fluorescent tag mCherry, treated axenic seedlings of *Trifolium pratense* with the labeled bacterium, and visualized the bacterium within epidermal cells and other leaf and root tissues using confocal microscopy (Figure 15 and Figure 16). The untreated *Trifolium pratense* seedlings did not show the internal presence of the tagged bacteria (Figure 15A). Sevigny et al. [84] isolated pseudomonads (*Pseudomonas fluorescens* and *Pseudomonas stutzeri*) from *Humulus lupulus* flowers (cones) and found the presence of nitrogenase genes in their genomes. We also isolated potential nitrogen-fixing species of *Massilia* and *Pantoea* from washes from *Humulus lupulus* cones (Table 3). There are thus data that suggest that the intracellular bacteria that may be observed in cells of leaves and bracts are growth-promoting bacteria that many investigators have isolated from plants as endophytes. For example, Benito et al. [80] showed that bacteria inoculated into roots may be transported to leaves. It may be that these microbes are more adapted to plants in terms of enzymatic capacities than most conspecifics from soil; it is also entirely possible that these bacteria can participate in the rhizophagy cycle to help the plant acquire nutrients from the soil [52]. Similarly, because trichomes and root hairs share similar morphologies and developmental programs [27], microbial associations (i.e., endophytism, parasitism, and symbiosis) observed in root hairs may also be anticipated features of trichomes. As root hairs have been discovered to inoculate the rhizosphere with microbes that participate in the rhizophagy cycle [13], so too may trichomes be inoculating the phyllosphere with microbes that benefit the plant defensively and nutritionally.

### 4.6. Evolutionary Considerations

**Figure 17:**
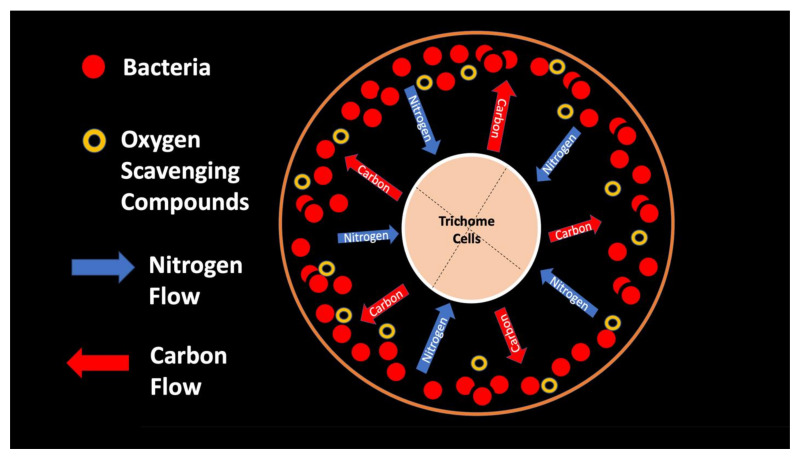
Suggested model of nitrogen transfer in glandular trichomes of *Cannabis sativa*: Carbon (sugars), produced by photosynthetic trichome stalk cells that bear the trichome head, are secreted (red arrows) from the central trichome cells (tan color) onto bacteria (red color). This stimulates growth and fuels nitrogen fixation by bacteria in the trichome. Trichome cells also secrete oxygen-scavenging compounds (yellow rings; e.g., cannabinoids) to scavenge atmospheric oxygen and reduce the inhibition of nitrogenase by oxygen. Trichome cells harvest nitrogen from bacteria by secreting superoxide onto bacteria, causing them to form wall-less protoplasts (L-forms) and stimulating them to secrete antioxidant nitric oxide (blue arrows), which combines with superoxide to form nitrate that may be absorbed by the central trichome cells.

From the experiments that we conducted, it is impossible to know whether plant trichomes evolved specifically as organs for nitrogen fixation or if, instead, trichomes are structures in which bacteria easily colonize and where some casual nitrogen transfer occurs between bacteria and plant cells. It is very likely that the endosymbiotic systems seen in leaves and bracts are less efficient than the more complex nitrogen-transfer endosymbiosis found in root nodules of legumes. Monocots are evolutionarily deeply rooted among seed plants [106]. It is notable that plants where we observed primarily or exclusively nuclear symbiosis in leaf parenchyma are monocots. These plants include *Agave* and *Hosta* spp. in the family Asparagaceae, *Vanilla* in the family Orchidaceae, and *Phragmites*, *Festuca*, and *Digitaria* in the family Poaceae, which have simple non-pitted trichomes. Grasses possess other modifications that may enhance the transfer of nutrients from bacteria to plant cells, making it likely that many other monocots (e.g., wheat, corn, and sorghum) possess this type of nuclear symbiosis. Epidermal cells of grasses develop lateral wall serrations or convolutions [107], and bacteria that emerge from the nucleus collect in these invaginations (likely in the periplasmic space) along the cell walls (Figure 3). In this location (predominantly in actively growing leaf cells), bacteria appear to interact chemically with the plant cell and appear to accumulate nitrogenous compounds (nitric oxide and nitrate) that may be absorbed directly into the plant cell. In plant cells without these lateral wall invaginations, clusters of bacteria usually accumulate at the ends of the cell in masses, where there is limited contact with the cytoplasm of the plant cell (White, unpublished data). It seems possible that grasses capitalized on this form of endosymbiosis by evolving the continuous formation of new leaves from basal meristems in tillers [107]. Many dicotyledonous plants also have trichomes that contain endosymbiotic bacteria, with pitted-filamentous trichomes being one form. In this type of trichome, bacteria form separate masses along the length of the hair, and this may result in the magnification of the chemical exchanges between plant trichome cells and endosymbiotic bacteria. Glandular trichomes may be a further improvement in the efficiency of nitrogen extraction. This hypothesis is largely based on our histochemical observations that glandular trichomes tended to stain densely for nitrogen forms (Figure 7). Glandular trichomes produce a suite of chemicals (flavonoids, terpenoids, oils, etc.) internally that could function as antioxidants to reduce the inhibitory effects of oxygen or control the proliferation and metabolism of bacteria [108,109,110,111]. The evolution of the glandular trichome with terpenoids as antioxidant compounds, or oxygen scavengers, may have increased nitrogen-fixing endosymbiosis. We propose a simple model for how glandular trichomes function (Figure 17), where glandular trichome chemistry functions to reduce oxygen around bacteria in trichomes to reduce oxygen inhibition of nitrogenase. Early developing glandular trichomes appear to show the presence of distinct clusters of rods with superoxide surrounding bacteria (Figure 8A), while more developed glandular trichomes show a mantle of what we interpret to be L-forms [15,16,37,52,57,70,73], with superoxide evident only at the interface between the eight central trichome cells and the mantle of bacteria (Figure 8B).

## 5. Conclusions

We found evidence to suggest that a diverse subset of plants cultivate endosymbiotic bacteria within non-photosynthetic cells of leaves and bracts and appear to extract nitrogenous nutrients from them. Our findings and hypotheses regarding endosymbiotic processes will need to be evaluated in greater depth by us and other labs. If future research continues to point to the extraction of nitrogen from intracellular microbes, this work could form an important foundation for the development of methods to grow crops using nitrogen provided by intracellular bacteria. Future research should focus on the following questions:

(1) Can nitrogen fixation within plant cells be confirmed?
(2) How are these endosymbioses regulated by the plant?
(3) What bacteria are involved in these endosymbioses?
(4) How robust are these endosymbioses?
(5) Are these endosymbioses lost in plants under cultivation?
(6) How might these endosymbioses function when plants are treated with nitrogen or other agrochemicals?
(7) Are there ways to treat plants to support or enhance these native endosymbioses in plant leaves?

Additionally, a host of evolutionary questions regarding the role of nitrogen extraction in plant diversification should be explored. For example, are comparable associations seen in non-seed plants (ferns and fern allies) and early seed plants (e.g., Gymnosperms)? The earliest plants to colonize land lacked extensive root systems; did they instead develop intracellular symbioses to extract nitrogen from endophytic bacteria? Is there fossil evidence for these types of associations in the early land plants?

## Figures and Tables

**Table 1:** Summary of histochemical experiments to assess chemical interactions between bacteria and plant cells in bracts and leaves of a variety of monocotyledonous and dicotyledonous plants ^1^.

Species	Family	Nuclear Symbiosis	Trichome Type		Ethylene			Reducing Sugars	Superoxide	Hydrogen Peroxide	Nitric Oxide	Nitrate
			F ^2^	G ^2^	AM	SM	PP	CS	NBT	DAB	FS	DS
*Agave boldinghiana*	Asparagaceae	√ ^3^			√			√				√
*Agave palmeri*	Asparagaceae	√					√		√		√	√
*Ailanthus altissima*	Simaroubaceae		√		√	√	√	√	√	√	√	√
*Apocynum cannabinum*	Apocynaceae			√								√
*Cannabis sativa*	Cannabaceae		√	√	√		√		√	√	√	√
*Celtis occidentalis*	Cannabaceae		√		√				√		√	√
*Citronella mucronata*	Cardiopteridaceae		√	√	√				√		√	√
*Digitaria sanguinalis*	Poaceae	√	√		√			√	√			√
*Digitaria ischaemum*	Poaceae		√		√							√
*Eupatorium altissimum*	Asteraceae		√		√				√			√
*Eutrochium maculatum*	Euphorbiaceae	√	√	√	√				√			√
*Festuca rubra*	Poaceae	√		√			√	√	√			√
*Glycine max*	Fabaceae		√		√		√	√	√	√	√	√
*Helianthus hirsutus*	Asteraceae		√									√
*Hedera helix*	Araliaceae		√		√							√
*Hosta plantaginea*	Asparagaceae	√			√		√	√	√		√	√
*Humulus lupulus*	Cannabaceae		√	√	√	√	√	√	√	√	√	√
*Lactuca sativa*	Asteraceae			√								√
*Lonicera japonica*	Caprifoliaceae		√	√	√		√		√	√	√	√
*Monotropa* *hypopitys*	Ericaceae		√		√			√	√			√
*Perilla frutescens*	Lamiaceae		√	√	√				√			√
*Phragmites australis*	Poaceae	√	√		√				√			√
*Rhus glabra*	Anacardiaceae		√	√	√		√		√		√	√
*Solanum dulcamara*	Solanaceae		√	√	√		√	√	√		√	√
*Solanum lycopersicum*	Solanaceae		√	√	√		√	√	√			√
*Solanum nigrum*	Solanaceae		√	√			√	√	√		√	√
*Solidago canadensis*	Asteraceae		√		√			√	√			√
*Stachys byzantina* ^4^	Lamiaceae		√									
*Thespesia populnea*	Malvaceae		√				√		√		√	√
*Vanilla phaeantha*	Orchidaceae	√			√			√	√		√	√
*Verbascum thapsus*	Scrophulariaceae		√				√		√		√	√
*Vigna radiata*	Fabaceae	√	√		√		√	√	√			√

^1^ Staining reagents are as follows: AM = ammonium molybdate; SM = sulfur monochloride; PP = potassium permanganate; CS = copper sulfate; NBT = nitroblue tetrazolium; DAB = diaminobenzidine tetrahydrochloride; FS = ferric (II) sulfate; DS = diphenylamine sulfate. ^2^ F = filamentous trichome, while G = glandular trichome. ^3^ A √ signifies a positive indication, while no √ indicates that no test was conducted. ^4^ No bacteria were observed in the long filamentous trichomes of *Stachys byzantina*.

**Table 2:** Results of 15 N assimilation experiments for *Agave boldinghiana*, *Ailanthus altissima*, *Hosta plantaginea*, *Humulus lupulus*, *Phragmites australis*, *Thespesia populnea*, and *Vanilla phaeantha*.

Plant Species	Treatment	Plant Organ	Delta 15N vs. Air ± SD (Number of Plants)	*p*-Value (Two-Tailed)/Group ID ^1^
*Agave boldinghiana*	15N_2_ enriched	Leaf #1 (youngest)	13.8 ± 12.6 (N = 3)	N/A ^2^
		Leaf #2	28.6 ± 9.8 (N = 3)	N/A
		Leaf #3	31.6 ± 5.4 (N = 3)	N/A
		Leaf #4	19.3 ± 8.8 (N = 3)	N/A
		Leaf #5 (oldest)	11.2 (N = 2)	N/A
		Roots	20.6 ± 28.3 (N = 2)	N/A
	Air control	Leaf #1 (youngest)	0.6 ± 0.4 (N = 2)	N/A
		Leaf #2	1.2 ± 0.8 (N = 2)	N/A
		Leaf #3	1.1 ± 0.4 (N = 2)	N/A
		Leaf #4	0.3 ± 1.0 (N = 2)	N/A
		Leaf #5 (oldest)	0.8 ± 0.4 (N = 2)	N/A
		Roots	4.2 ± 0.5 (N = 2)	N/A
*Agave palmeri*	15N_2_ enriched	Seedling leaves	74.4 ± 21.3 (N = 3)	N/A
	Air control	Seeding leaves	3.9 ± 0.8 (N = 2)	N/A
*Ailanthus altissima*	15N_2_ enriched	Leaflets	1017.6 ± 3.5 (N = 2)	N/A
		Leaf rachis	673.3 (N = 1)	N/A
	Air control	Leaflets	3.38 (N = 1)	N/A
		Leaf rachis	3.48 (N = 1)	N/A
*Festuca rubra*	15N_2_ enriched	Seedling shoots (leaf blades and sheaths)	236.7 ± 102.5 (N = 4)	N/A
	Air	Seedling shoots (leaf blades and sheaths)	1.3 ± 1.0 (N = 2)	N/A
*Hosta plantaginea*	15N_2_ enriched	Leaves	674.5 ± 369.4 (N = 4)	0.03528 (Leaves in air vs. N15) ^3^
				0.049734 (Leaves vs. roots in N15)
		Roots	87.5 ± 56.7 (N = 4)	0.052163 (Roots in air vs. N15)
				0.96609 (Leaves vs. roots in air)
	Air control	Leaves	−1.4 ± 1.8 (N = 3)	
		Roots	−1.3 ± 1.9 (N = 3)	
*Humulus lupulus*	15N_2_ enriched	Inflorescences	2539.6 ± 1329.7 (N = 3)	0.6283 (Inflorescences vs. leaves/stems in N15) ^3^
		Leaves and stems	2206.8 ± 1691.8 (N = 3)	0.6283 (Inflorescences vs. leaves/stems in N15)
	Air control	Inflorescences	13.5 (N = 1)	N/A
		Leaves and stems	13.1 (N = 1)	N/A
*Lonicera japonica*	15N_2_ enriched	Leaves	52 ± 16.0 (N = 6)	0.000587 (Leaves in air vs. N15)
	Air control	Leaves	1.6 ± 0.1 (N = 3)	
*Phragmites australis*	15N_2_ enriched	Leaves	23.0 ± 2.0 (N = 3)	No significant differences between plant parts (Leaves/stems/roots in N15)
		Stems	21.0 ± 7.1 (N = 3)	
		Roots	15.5 ± 4.7 (N = 3)	
	Air control	Leaves	4.8 (N = 1)	N/A
		Stems	1.8 (N = 1)	N/A
		Roots	2.1 (N = 1)	N/A
*Thespesia populnea*	15N_2_ enriched	Leaf #1 (youngest)	545.2 ± 179.0 (N = 4)	A (Comparison of plant parts in N15) ^4^
		Leaf #2	650.2 ± 66.9 (N = 4)	A
		Leaf #3	488.1 ± 79.0 (N = 4)	AB
		Leaf #4 (oldest)	441.1 ± 103.2 (N = 4)	AB
		Roots	248.9 ± 89.1 (N = 4)	B
	Air control	Leaf #1 (youngest)	3.9 (N = 1)	N/A
		Leaf #2	4.4 (N = 1)	N/A
		Leaf #3	4.9 (N = 1)	N/A
		Leaf #4 (oldest)	4.2 (N = 1)	N/A
		Roots	2.6 (N = 1)	N/A
*Vanilla phaeantha*	15N_2_ enriched	Leaf #1 (youngest)	63.9 ± 14.4 (N = 8)	A (Comparison of plant parts in N15) ^4,5^
		Leaf #2	40.9 ± 20.8 (N = 8)	AB
		Leaf #3	24.9 ± 12.6 (N = 8)	BC
		Leaf #4 (oldest)	20.2 ± 18.7 (N = 5)	BC
		Roots	6.8 ± 1.6 (N = 4)	C
	Air control	Leaf #1 (youngest)	3.9 (N = 1)	N/A
		Leaf #2	4.2 (N = 1)	N/A
		Leaf #3	2.3 (N = 1)	N/A
		Leaf #4 (oldest)	3.4 (N = 1)	N/A
		Roots	2.3 (N = 1)	N/A

^1^*t*-test (unequal variance; Excel Data Analysis Toolpak) comparing plant parts of treated (N15) vs. untreated (air) plants. ^2^ N/A indicates that statistical analysis could not be conducted due to insufficient replication. ^3^ Paired *t*-test (Excel Data Analysis Toolpak) comparing plant parts within N15 treatment group in *H. lupulus*. ^4^ Repeated measures ANOVA with Tukey’s HSD post hoc comparisons (vassarstats; alpha = 0.05; groups with same letter are not statistically different from each other). Comparisons made between plant parts within N15 group. ^5^ A subset of the data (N = 3) was used for analysis due to missing data for some parts of some plants.

## Data Availability

All data are in the article.
